# miR-24-2 controls *H2AFX *expression regardless of gene copy number alteration and induces apoptosis by targeting antiapoptotic gene *BCL-2*: a potential for therapeutic intervention

**DOI:** 10.1186/bcr2861

**Published:** 2011-04-04

**Authors:** Niloo Srivastava, Siddharth Manvati, Archita Srivastava, Ranjana Pal, Ponnusamy Kalaiarasan, Shilpi Chattopadhyay, Sailesh Gochhait, Raina Dua, Rameshwar NK Bamezai

**Affiliations:** 1 National Centre of Applied Human Genetics, School of Life Sciences, Jawaharlal Nehru University (JNU), New Mehrauli Road, Saraswatipuram, New Delhi 110 067, India; 2 School of Biology and Chemistry, Shri Mata Vaishno Devi University, Kakriyal, Katra, Jammu and Kashmir 182320, India; 3 International Centre for Genetic Engineering and Biotechnology, Aruna Asaf Ali Marg, New Delhi 110067, India

## Abstract

**Introduction:**

New levels of gene regulation with microRNA (miR) and gene copy number alterations (CNAs) have been identified as playing a role in various cancers. We have previously reported that sporadic breast cancer tissues exhibit significant alteration in *H2AX *gene copy number. However, how CNA affects gene expression and what is the role of miR, miR-24-2, known to regulate *H2AX *expression, in the background of the change in copy number, are not known. Further, many miRs, including miR-24-2, are implicated as playing a role in cell proliferation and apoptosis, but their specific target genes and the pathways contributing to them remain unexplored.

**Methods:**

Changes in gene copy number and mRNA/miR expression were estimated using real-time polymerase chain reaction assays in two mammalian cell lines, MCF-7 and HeLa, and in a set of sporadic breast cancer tissues. *In silico *analysis was performed to find the putative target for miR-24-2. MCF-7 cells were transfected with precursor miR-24-2 oligonucleotides, and the gene expression levels of *BRCA1, BRCA2, ATM, MDM2, TP53, CHEK2, CYT-C, BCL-2, H2AFX *and *P21 *were examined using TaqMan gene expression assays. Apoptosis was measured by flow cytometric detection using annexin V dye. A luciferase assay was performed to confirm BCL-2 as a valid cellular target of miR-24-2.

**Results:**

It was observed that *H2AX *gene expression was negatively correlated with miR-24-2 expression and not in accordance with the gene copy number status, both in cell lines and in sporadic breast tumor tissues. Further, the cells overexpressing miR-24-2 were observed to be hypersensitive to DNA damaging drugs, undergoing apoptotic cell death, suggesting the potentiating effect of mir-24-2-mediated apoptotic induction in human cancer cell lines treated with anticancer drugs. BCL-2 was identified as a novel cellular target of miR-24-2.

**Conclusions:**

mir-24-2 is capable of inducing apoptosis by modulating different apoptotic pathways and targeting *BCL-2*, an antiapoptotic gene. The study suggests that miR-24-2 is more effective in controlling *H2AX *gene expression, regardless of the change in gene copy number. Further, the study indicates that combination therapy with miR-24-2 along with an anticancer drug such as cisplatin could provide a new avenue in cancer therapy for patients with tumors otherwise resistant to drugs.

## Introduction

Copy number variations (CNVs) are ubiquitous in nature and have been identified in diverse species, including humans [1], monkeys [2], rats [3], mice [4] and *Drosophila *[5]. Advancement in DNA array technology has led to the discovery of CNVs that are now believed to cover at least 10% of the total human genome [6]. In a short span of time since their discovery, CNVs have been characterized and shown to play a role in a number of human diseases, including cancers. Among the DNA repair genes, changes in gene copy numbers of *BRCA2 *and *H2AFX *have been shown to be associated with ovarian cancer [7] and breast cancer [8], respectively. Although the importance of CNVs (in germline cells) [9] or alterations (in somatic cells) [7,10] has been uncovered in recent years, their molecular and cellular consequences remain to be understood completely.

H2AX is a variant of histone H2A, and is rapidly phosphorylated at serine 139 by members of the phosphatidyl inositol 3-kinase family of kinases [11,12] in response to different cellular stressors, such as DNA double-stranded breaks, osmotic stress, replication blockage and hyperthermia [13-18]. In the past decade, H2AX has generated much scientific interest, not only because of its functional enormity but also because of its localization in highly vulnerable cytogenetic regions, such as 11q23.3, which is known to undergo frequent alteration in most human cancers, including breast cancer [19-23]. The *H2AX *gene is not essential, but its absence shows increased genomic instability and sensitivity to DNA damaging agents [24,25]. Recently, the microRNA (miR) miR-24-2 has been identified as a regulator of *H2AX *gene expression [26]. A large number of studies have signified the important role of miR in cell proliferation and apoptosis [27,28]. Some miRs, such as miR-29b and miR-15-16, modulate the apoptotic pathway, whereas a few others, including miR-24, let-7/miR-98 and miR-17-92 have been shown to affect both the apoptotic and cell proliferation pathways [29].

In the present study, we observed that regardless of alterations in gene copy number, the expression of *H2AX *is regulated by miR-24-2. MCF-7 and HeLa cells were utilized as model cell lines, since the two showed differential *H2AFX *gene copy numbers [8] and the findings were then confirmed in a representative set of breast carcinoma samples. miR-24-2 has been reported to modulate the cell's apoptotic response; however, the only gene target identified with respect to apoptotic function is Fas-associated factor 1 (FAF1) [30]. Our study identifies the antiapoptotic gene *BCL-2 *as a novel biological target of miR-24-2 and suggests that overexpression of miR-24-2 induces apoptosis by downregulating the expression of genes such as *BCL-2, MDM-2, H2AFX *and *P21*.

## Materials and methods

### Cell culture

MCF-7 and HeLa cells were cultured in RPMI 1640 medium (Sigma, St. Louis, MO, USA). Media were supplemented with 10% fetal bovine serum, 1 mmol/l L-glutamine and 50 μg/ml penicillin/streptomycin.

### Tumor samples

Tissue samples from patients with sporadic ductal breast carcinoma were obtained from Dharamshilla Cancer Hospital and Rajiv Gandhi Cancer Research Institute, Delhi, India. Informed written consent following the Indian Council of Medical Research norms was obtained from all individuals, and the ethics committee of Jawaharlal Nehru University approved the study. Clinicopathological details were also obtained from the patients with their consent.

### Determination of H2AX copy number

The relative change of H2AX copy number between normal and tumor pairs or different cell lines was determined by real-time polymerase chain reaction (RT-PCR) assay following the comparative threshold cycle (*C*_t_) method [31]. The TaqMan assay(Applied Biosystems, USA) used for H2AX was Hs01573336_s1. The target gene and the reference gene (RNase P) were amplified separately using the ABI PRISM 7000 Sequence Detection System (PE Applied Biosystems, Foster City, CA, USA). PCR was performed in a total volume of 25 μl in each well, which contained 12.5 μl of TaqMan Universal MasterMix (PE Applied Biosystems), 25 ng of genomic DNA and a 12.5 picomoles per liter concentration of each primer. PCR conditions included an initial denaturation step of 95°C for 10 minutes, followed by 40 cycles at 95°C for 15 seconds and 60°C for 1 minute. All of the reactions were carried out in duplicate, and a negative control with no template was kept with every PCR run. For all PCR assays, *C*_t _numbers were established by using SDS 1.1 RQ software (Applied Biosystems), and the copy number, normalized against a reference gene (RNase P), and the calibrator (normal sample of the respective pair) were determined by using the formula 2^-ΔΔ*C*t^. A twofold increase or decrease in the copy number of H2AX in tumor samples in comparison to the corresponding normal sample within the pair was considered as amplification or deletion, respectively.

### RNA isolation and quantitative RT-PCR

Total RNA was extracted from tumor samples and cell lines by using TRIzol reagent (Sigma) according to the manufacturer's instructions. RNA quality from each sample was determined by the A260/A280 absorbance ratio and by electrophoresis on 1.2% agarose formaldehyde gel. Quantities of 1.0 to 2.0 mg of total RNA were reverse transcribed into single-stranded cDNA using the Omniscript Reverse Transcriptase kit (Qiagen, Hildane, Germany). The commercially available TaqMan Gene Expression Assay system (Applied Biosystems) was used for quantitating transcription levels of H2AX, ATM, TP53, CHK-2, Bcl-2, p21, MDM2, BRCA1, BRCA2 and CYT-C. Quantitative RT-PCR was carried out using an ABI Prism 7000 Sequence Detection System (Applied Biosystems). *C*_t _numbers were established by using SDS 1.1 RQ software (Applied Biosystems), and Δ*C*_t _values were determined (Δ*C*_t _= *C*_t _of target gene - *C*_t _of internal control) as raw data for gene expression. All the reactions were carried out in duplicate, and fold changes in gene expression were determined by using the formula 2^-ΔΔ*C*t^. geNorm software [32] was used to establish the two most stable internal control genes (*MRPL19 *and *PUM1*) from a group of four endogenous controls (*ACTIN, GAPDH, PUM1 *and *MRPL19*), followed by the calculation of the normalization factor for each tissue sample.

### Confocal microscopy and image capturing

Cells were grown on coverslips in Dulbecco's modified Eagle's medium. At 70% confluence, the cells were fixed in 4% paraformaldehyde for 30 minutes at room temperature. The cells were then washed in phosphate-buffered saline (PBS) thrice at 5-minute intervals and processed for immunostaining. The cells were incubated in blocking buffer for 1 hour at 37°C before overnight incubation with rabbit polyclonal primary antibodies (anti-H2AX and anti-γ-H2AX; Bethyl Laboratories, USA) at 4°C and diluted (1:500) in blocking buffer. Following 15-minute washes in PBS + 0.1% Triton X-100 (PBST) thrice, the signals were detected after incubation with chicken anti-rabbit Alexa Fluor 488 ( Invitrogen, Bangalore, India) diluted 1:1,000 at 37°C for 2 hours. After 15-minute PBST washes thrice, the cells were counterstained with propidium iodide (PI) along with RNase (10 μg/ml PI and 200 μg/ml RNase A) treatment for 7 to 10 minutes at 37°C and mounted in DABCO (Sigma).

### Image capturing

Stained cells were observed with a Nikon TE 2000E microscope (Nikon, Japan) equipped with a ×60/1.4 NA Plan-Apochromat (Carl Zeiss, NY, USA) DIC objective. PI was excited at 543 nm with He-Ne laser and Alexa Fluor 488 at 488 nm with an argon ion laser. The emissions were recorded through an emission filter set 515/30, 605/75. Images were acquired sequentially to avoid bleed-through, with a scanning mode format of 512 × 512 pixels. The transmission and detector gains were set to achieve the best signal-to-noise ratios, and the laser powers were tuned to limit bleaching of fluorescence. The refractive index of the immersion oil used was 1.515 (Nikon). All settings were rigorously maintained for all experiments.

All images were qualitatively assessed using Image Pro Plus version 6.0 software (Media Cybernetics, Bethesda, MD, USA). All the images were stored in Tiff RGB 24 format. To reduce the unwanted ground noise generated by the photomultiplier signal amplification, the images were treated with two-dimensional filters (Gaussian and sharpening filtering).

### *In silico *analysis

Many computational target prediction software platforms have been developed to identify the miR binding sites in 3'UTR of the of the gene transcripts. To avoid spurious prediction, four widely used software platforms, PicTar [33], miRBase Targets version 5 [34], TargetScan [35] and microRNA[36], were used to perform the target prediction. *H2AFX *transcription was predicted as a good target for hsa-miR-24-2 by all four prediction software types, and miR-24-2 was found to have two possible binding sites in the 3'UTR of H2AX mRNA (Table S1 in Additional file 1). Microrna.org (miRanda algorithm) predicted *BCL2*, while TargetScan predicted *MDM2*, as a target gene for miR-24-2. However, transcripts of *TP53, P21 *and *CYT-C *were not detected by any of the software platforms as targets of miR-24-2.

### Transfection and miR assay

Transfection was performed using ESCORT transfection reagent (Sigma). Synthetic pre-miR-24-2 oligonucleotides (Ambion, Austin, TX, USA) or antagomir (Ambion) were transfected at a final concentration of 50 nmol/l. Transfection with a pre-miR negative control oligonucleotide (Ambion, PM 17001) was always used as a negative control. Cells were harvested 48 hours after transfection, and RNA was obtained using the *mir*Vana™ miRNA Isolation Kit (Ambion). The quantity and quality of RNA were analyzed by Nanodrop (NanoDrop Technologies, Wilmington, DE, USA) using 260/280 nm and gel analysis. TaqMan microRNA assays (Applied Biosystems) that include specific RT primers and TaqMan probes were used to quantify the expression of mature miR-24-2 (Assay ID 002441; PN 4427975), and RNU 44 (Assay ID 001094; PN 4427975) was used for normalization.

### Apoptosis assay

Apoptosis was measured by the flow cytometric detection of phosphatidylserine externalization using APC Annexin V staining (BD Biosciences, MD, USA). MCF-7 cells, after transfection with pre-miR-24-2 and pre-miR negative controls, were treated with 200 μmol/l cisplatin for 24 hours (Sigma, Louis, MO, USA) and 25 mmol/l for 20 minutes H_2_O_2 _(Merck, NJ, USA). The cells were harvested and processed for APC Annexin V staining as per the manufacturer's protocol (BD Biosciences). Briefly, cells were washed twice with binding buffer (10 mmol/l (4-(2-hydroxyethyl)-1-piperazineethanesulfonic acid, 140 mmol/l NaCl and 5 mmol/l CaCl_2_, pH 7.4) and stained with APC-conjugated annexin V for 15 minutes at room temperature, followed by flow cytometric analysis using the Becton Dickinson FACSCalibur (Franklin Lakes, NJ, USA). The extent of apoptosis was quantified as the percentage of annexin V-positive cells.

### Luciferase assay

Luciferase assay was performed to confirm the interaction of miR-24-2 with the predicted binding sites of the genes. The miR-24-2 predicted binding sites in the 3'UTR of the *BCL2 *and *H2AFX *genes were amplified by using specific primers (Table S3 in Additional file 1), and the amplicons were cloned at the 3'UTR of luciferase gene in pGL3 control vector. The positive clones were confirmed by sequencing and then used for the luciferase assay. The assay was performed in two different mammalian cell lines, HepG2 and MCF-7, simultaneously. Briefly, cells were seeded in 12-well plates, and, after 24 hours of growth, they were transfected with specific sets of plasmid mix (pEP-miR-24-2 + pGL3-*BCL2/H2AFX*+ pRL-TK) using ESCORTS reagent (Sigma). A pEP-miR-24-2 vector (Cell Biolabs, San Diego, CA, USA) was used to overexpress miR-24-2 in cells. After 48 hours of transfection, cells were assayed to measure firefly and Renilla luminescence using the luciferase kit (Promega, Madison, WI, USA). The ratio of firefly reporter and Renilla control reporter in the presence of miR-24-2 was calculated and then used to define the change in the expression of firefly reporter in the co-presence of predicted binding sites of specific genes and in the overexpression of miR-24-2.

## Results

### *H2AFX *gene copy number and transcript expression in MCF-7 and HeLa cells

**Figure 1:**
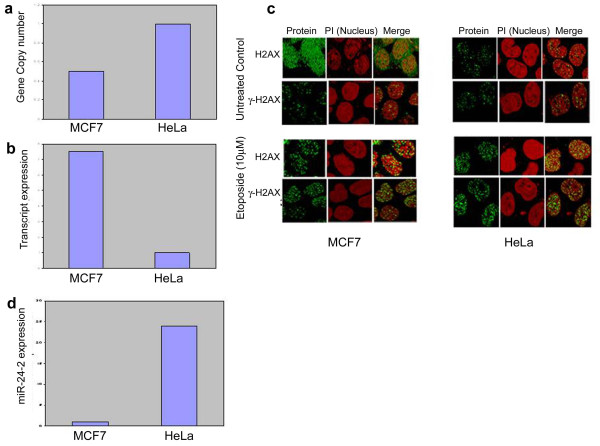
**Comparison of *H2AFX *status in MCF-7 vs. HeLa cells**. **(a) **Real-time polymerase chain reaction analysis of gene copy number between the two cell lines using RNase P as an endogenous control. **(b) **Comparison of transcript expression using TaqMan assay for H2AX (Hs01573336_s1). **(c) **Confocal images of subcellular localization of H2AX and γ-H2AX before and after etoposide (10 μmol/l) treatment. **(d) **Fold difference in hsa-miR-24-2 expression using RNU 44 (Assay ID 001094; PN 4427975) for normalization. PI, propidium iodide.

*H2AFX *gene copy number as measured by RT-PCR assay using TaqMan chemistry revealed twofold deletions in MCF-7 cells compared to HeLa cells (Figure 1a). To ascertain whether this change in copy number brought about a corresponding change in gene expression, RT-PCR analysis of the transcripts was performed. A sevenfold higher expression was observed in MCF-7 cells compared to the transcription level in HeLa cells in a simultaneous study (Figure 1b). This noncorrespondence of expression with the CNA was paradoxical. Further confirmation of these observations in the two cell lines, with a loss (MCF-7) but high transcription expression and gain (HeLa) with a relatively low expression, was carried out in *in situ *protein level expression in a confocal study (Figure 1c). The presence of an increased amount of the unphosphorylated form of H2AX in MCF-7 nuclei and cytosol corroborated with the higher expression of transcripts, despite low CNA in the *H2AFX *gene. To establish whether the increased H2AX staining was due to an inherent DNA damage status of the MCF-7 cells used, two approaches were adopted. First, serine 139 phosphorylation of the H2AX protein (γ-H2AX) serves as a very good marker of DNA damage, and therefore we used phosphorylated H2AX antibodies to detect the difference between phosphorylated and unphosphorylated forms of H2AX. Second, we assessed the induction of γ-H2AX after exposure to etoposide, a potent DNA damaging drug. The confocal analysis (Figure 1c) revealed that both the cell lines with two different features of CNA and the expression profiles at the transcript and protein levels showed no difference in their response to DNA damage at both the endogenous (γ-H2AX staining in untreated control cells) and exogenous levels (γ-H2AX staining after etoposide treatment), suggesting that the inherent tendency of CNA and corresponding expression were independent of the DNA damage response (DDR), which was equal. We nevertheless were still confronted with the problem of noncorrespondence of the *H2AFX *gene copy number with its transcript level and therefore analyzed the expression of miR-24-2, another regulatory control for *H2AFX *gene expression. A bioinformatics search for possible miR regulation using four bioinformatics tools (miRanda[36], microCosm targets[37], PicTar[33] and Target Scan[35]) indicated miR-24-2 as the most likely potential regulator of the *H2AFX *gene (Table S1 in Additional file 1). Also, during the course of this study, a report experimentally validated the miR-24-2-mediated downregulation of H2AX in terminally differentiated mammalian cells [26].

### miR-24-2 expression in the two model cell lines

We examined the expression of miR-24-2 by RT-PCR analysis that uncovered 14-fold higher miR-24-2 levels in HeLa cells than in MCF-7 cells (Figure 1d). This observation provided an explanation for the ambiguity observed in experiments between gene copy number and transcript status of MCF-7 and HeLa cells. It is likely that the expression in MCF-7 cells was high because of the low level of miR-24-2 present in these cells, resulting in lower transcriptional degradation of H2AX mRNA and therefore not corresponding with the CNA status. Higher levels of miR-24-2 in HeLa cells, on the other hand, allowed destabilization of a larger fraction of the synthesized mRNA, resulting in the detection of lower expression of the transcripts.

### Replication of the study in a representative set of breast carcinoma samples

**Figure 2:**
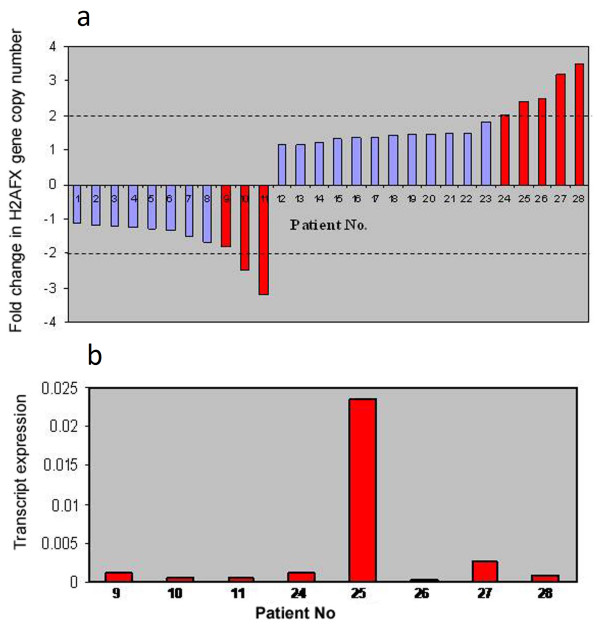
**Noncorrespondence of *H2AFX *gene copy number and transcript expression in patients with sporadic breast cancer**. **(a) ***H2AFX *gene copy number alteration in patients with sporadic breast cancer. Patients 9 to 11 show deletion, whereas patients 24 to 28 show amplification. A twofold change and above was considered deletion or amplification. **(b) ***H2AFX *transcript expression in patients with altered gene copy number. Note the noncorrespondence of gene copy number and transcript expression with the exception of patient 25.

**Figure 3:**
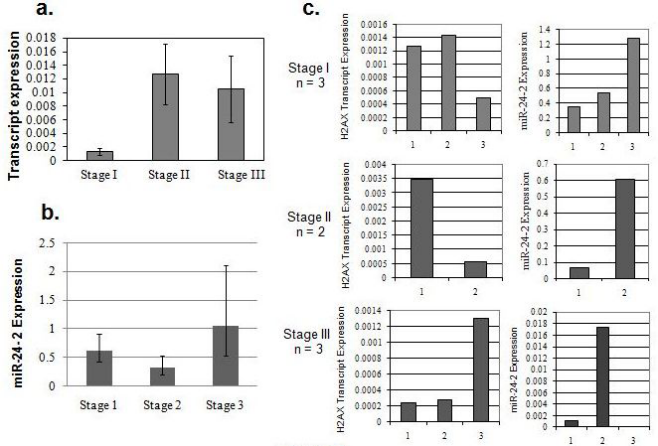
**Analysis of microRNA (miR) hsa-miR-24-2 expression level in sporadic breast cancer samples of different stages (stages I, II and III) and its correlation with H2AX transcript expression**. **(a) **H2AX transcript expression. **(b) **miR-24-2 expression. **(c) **Individual patient-wise comparison of H2AX transcript and miR-24-2 expression in representative breast tumor samples of stage I (*n *= 3), stage II (*n *= 2) and stage III (*n *= 3).

The analysis of the two cell lines, MCF-7 and HeLa, suggested that alteration in *H2AFX *gene copy number does not directly regulate its expression; instead, the expression is more strongly controlled by a miR, hsa-miR-24-2. To corroborate the above observations, we repeated the same analysis in sporadic breast tumor samples that also exhibited alteration in *H2AFX *gene copy number. Breast cancer samples (36 pairs) belonging to stages I, II and III showed an alteration in gene copy number in 22% (8 of 36) of cases, which involved both amplification and deletion when compared to normal samples. The deletion accounted for 8.3% (3 of 36) of the cases and amplification in about 13.8% (5 of 36) of samples (Figure 2a). The tumors from these samples were subjected to real-time transcript analysis using TaqMan chemistry. geNorm software was used to establish the two most stable internal control genes (*MRPL19 *and *PUM1*) from a group of four endogenous controls (*ACTIN, GAPDH, PUM1 *and *MRPL19*), followed by the calculation of the normalization factor for each tissue sample (Table S2 in Additional file 1). It was observed that of eight samples showing genomic copy number alteration (CNA), only one (sample 25) showed correspondence with the transcript level. Seven other samples with either deletion or amplification did not show any parallel between the gene CNA and transcriptional status (Figure 2b). As observed in cell lines, the studied tumor samples also showed a noncorrespondence between CNA and transcript expression. To examine whether *H2AX *gene expression in tumor tissues also corresponds negatively with miR-24-2 expression, the paired tumor samples were examined for miR-24-2 expression in 33 tumor samples and 13 normal breast tissue samples. miR-145, a known miR that is downregulated in breast cancers, and RNU-44 as an endogenous miR, were used as controls. As expected, miR-145 was downregulated in all the cancer stages, but miR-24-2 showed differential status with respect to different stages of tumors (Figures S1a and S1b in Additional file 2). Compared to corresponding normal tissue samples, miR-24-2 was low in tumors and was relatively higher in stage I and lower in stages II and III tumor tissue samples, with an inverse relation between mir-24-2 and H2AX mRNA expression (Figures 3a and 3b). The expression of both the *H2AX *gene and miR-24-2 in individual patients with tumors at different stages was again observed to have an inverse relation (Figure 3c), confirming miR-24-2 as a strong regulator of H2AX in *in vivo *sporadic breast tumors.

### *In vitro *overexpression of miR-24-2 in MCF-7 cells and modulation of apoptotic response

**Figure 4:**
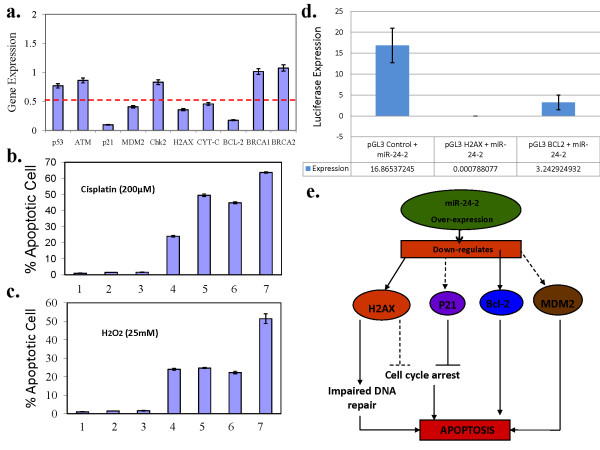
**miR-24-2 overexpression and its effect on gene expression and cell proliferation**. **(a) **Effect of miR-24-2 upregulation on gene expression profile of MCF-7 cells for *TP53, ATM, P21, MDM2, CHEK2, H2AFX, CYT-C, BCL-2, BRCA1 *and *BRCA2*. **(b) **miR-24-2 overexpressing MCF-7 cells treated with cisplatin (200 μmol/l) and assayed for apoptotic cell death using annexin V staining and fluorescence-activated cell sorting analysis. **(c) **miR-24-2-overexpressing MCF-7 cells treated with H_2_O_2 _(25 mmol/l) and analyzed for apoptosis. (1) Negative control (unstained MCF-7 cells), (2) MCF-7 control cells, (3) MCF-7 + mock transfection, (4) MCF-7 + miR-24-2 transfection, (5) MCF-7 + H_2_O_2_/cisplatin, (6) MCF-7 + mock transfection + H_2_O_2_/cisplatin and (7) MCF-7 + miR-24-2 transfection + H_2_O_2_/cisplatin. The extent of apoptosis was quantified as percentage of annexin V-positive cells. Error bars indicate standard deviation. **(d) **Luciferase expression in MCF-7 cells overexpressing miR-24-2 and transfected with pGL3 control vector or vector harboring the predicted miR-24-2 binding site present in 3'UTR of *H2AFX/BCL-2 *genes. **(e) **Proposed model of miR-24-2-mediated apoptotic induction. Following overexpression of miR-24-2, the mRNA expression of key apoptotic (*BCL-2 *and *MDM2*)/DNA damage response genes (*H2AFX *and *P21*) is downregulated. While downregulation of H2AX would lead to impaired DNA repair and loss of cell-cycle arrest, reduced expression of p21 prevents entry into cycle arrest pathway and instead signals the apoptotic pathway. Reduced *BCL-2 *and *MDM2 *expression is capable of directly inducing the apoptotic pathways leading to cell death.

To study the effect of miR-24-2 overexpression on gene expression, MCF-7 cells were transfected with precursor miR-24-2 oligonucleotides, and the overexpression of miR-24-2 was verified by real-time TaqMan assay (Figure S2 in Additional file 2). Downregulation of *H2AX *expression in miR-24-2-overexpressing cells confirmed *H2AX *as a cellular target of miR-24-2 (Figure 4a). Interestingly, overexpression of miR-24-2 also resulted in increased apoptotic cell death as assayed by annexin V staining. This effect of miR-24-2 overexpression was further evident in response to the DNA damaging drug cisplatin (200 μmol/l), as well as to hydrogen peroxide (25 mmol/l), as compared to untransfected and negative precursor oligonucleotide (AM17110; Ambion) transfected controls (Figures 4b and 4c). The observed hypersensitivity to drugs, increased apoptosis and decreased H2AX expression in cells over-expressing miR-24-2 indicated a possible role of H2AX in regulating apoptosis. In this context, it is interesting to note that phosphorylation of tyrosine 142 residue of H2AX has been shown to modulate a cell's decision to enter into the apoptotic or survival pathway [38,39]. Also, it could be possible that in addition to H2AX, miR-24-2 regulates other key genes of the apoptotic pathway. To test this possibility, we analyzed the expression of key apoptotic and DDR genes (*BRCA1, BRCA2, ATM, MDM2, TP53, CHEK2, CYT-C, BCL-2 *and *P21*) in cells after overexpression of miR-24-2 (Figure 4a). The transcript expression of *H2AFX, BCL-2, MDM2 *and *P21 *were significantly reduced and therefore suggested that BCL-2, MDM2 and p21 could possibly be the cellular targets of miR-24-2. Intriguingly, the bioinformatics analysis also revealed the presence of miR-24-2 binding sites in BCL-2 and MDM2 mRNA besides having two binding sites in H2AFX mRNA (Figure S3 in Additional File 2), however, the binding site could not be identified in P21 mRNA. We further tested the gene expression in MCF-7 cells transfected with miR-24-2-specific antagomirs, and it was observed that inhibiting miR-24-2 expression resulted in significantly enhanced expression of *BCL-2 *and *H2AFX *compared to mock transfected control (Figure S7 in Additional file 2). The study therefore suggested BCL-2 as a possible novel cellular target of miR-24-2 and confirmed H2AX regulation by miR-24-2 in proliferating cell lines and in tumor samples.

### miR-24 regulates *BCL-2 *gene by binding to the predicted 3'UTR sites

*BCL-2 *is a known antiapoptotic gene. To confirm the presence of a putative binding site for miR-24-2 within the 3'UTR region of the *BCL-2 *gene, the specific primers flanking the binding sites were designed and the resulting amplicon was cloned into the 3'UTR region of the luciferase gene of the reporter vector pGL3 (pGL3/*BCL-2*). H2AX, known to be regulated by miR-24-2, was used as a positive control for the luciferase assay. The luciferase reporter vectors were co-transfected into MCF-7 and HepG2 cells with pEP-miR-24-2 vector. Subsequently, luciferase activity was measured. It was observed that overexpression of miR-24-2 was able to decrease the luciferase activity of the reporter vector containing *BCL-2/H2AFX *miR-24-2 binding sites (Figure 4d). These data showed that miR-24-2 could downregulate its targets, *BCL-2 *and *H2AFX*, by binding to the predicted binding sites and hence provide a mechanistic insight into the apoptotic induction caused by its overexpression.

## Discussion

The observations made in this study suggest that the *H2AFX *gene undergoes CNA in patients with sporadic breast cancer, as well as in studied cancer cell lines; however, the expression status does not correspond with the CNA status. Two recent studies in rats and mice at a genome-wide scale have described the effect of CNVs on gene expression, exhibiting negative correlation in 2% to 15% of the genes with their expression [3,4]. We provide evidence for one of the possible mechanisms of such a nonconcordant relation between expression and the number of gene copies based on specific miR regulation of expression. One such miR, hsa-miR-24-2, that has been reported to be a strong regulator of *H2AX *expression [24] was confirmed in our study, both in cell lines and in sporadic breast tumor samples, irrespective of CNA. Interestingly, it was observed that overexpression of miR-24-2 downregulated the transcript expression of *H2AFX *alongwith *BCL-2, MDM2*and *P21*, with a corresponding increase in apoptotic cell death, suggesting an adoption of a new paradigm in therapeutic designs to overcome apoptotic resistance in cancer cells. The role of miR-24-2 in regulation of apoptosis has been shown by a few studies, but the regulation of pro- or antiapoptotic genes by this miR is not known, except for FAF1 [30]. Our study provides the mechanistic insight into the apoptotic induction mediated by miR-24-2 and identifies BCL-2 as the novel cellular target of miR-24-2 (Figure 4e). We propose that while downregulation of H2AX results in impaired DNA repair, channeling the cells into the apoptotic pathway, downregulated BCL-2, encoding an integral outer mitochondrial membrane protein and known to block the apoptotic death in a variety of cell systems [40], could contribute further to apoptotic cell death [41]. It has been shown that H2AX is required for the p53/p21 pathway [42], and it is expected that the lower level of H2AX expression could prevent the cells from cell cycle arrest and promote induction of apoptosis. We have also observed that *MDM2 *and *P21 *possibly could emerge as other key genes that promote apoptotic induction and whose expression is modulated by miR-24-2, either directly or indirectly. This, however, would require experimental confirmation through reporter gene assays in future studies. Nevertheless, on the basis of our findings, we propose that miR-24-2 is a strong inducer of apoptotic pathway in MCF-7 cells by controlling the expression of important genes involved in apoptotic regulation. MDM2 and p21 are known as key players in regulating the p53 response to induce apoptosis or growth arrest [43]. MDM2 acts as an oncoprotein that promotes cell survival and cell cycle progression by inhibiting the p53 tumor suppressor protein [44]. Also, low levels of MDM2 have been shown to induce the transcription of proapoptotic genes and the translocation of p53 from nucleus to mitochondria, resulting in apoptosis [45]. p21 is a cyclin-dependent kinase inhibitor (CDKN1A) and functions as a regulator of cell cycle progression to G1 in response to p53 checkpoint pathway [46]. Its role in apoptosis is not very clear, but the possibility is that low expression of p21 would prevent the cells from p53/p21-mediated cell cycle arrest pathway and result in induction of apoptosis [47]. Since p21 transcripts do not have a miR-24-2 binding site, we surmise that the expression of p21 gets reduced as a result of secondary effect and could possibly be a secondary target of miR-24-2 [48]. Interestingly, we have also tested the apoptotic potentiating activity of miR-24-2 in the presence of a mitotic inhibitor drug, docetaxel, and observed a significant increase in cell death in MCF7 cells that have received combination treatmentof docetaxel (2 nmol/l) and miR-24-2 over-expression (500 ng of pEP-miR-24-2) as compared to MCF7 cells that have received docetaxel treatment or mir-24-2 over-expression alone (data not shown). We propose that the lower expression of these genes as a result of miR-24-2 overexpression could independently, or in association with other proteins, target different apoptotic pathways and provide an alternative window for effective tumor cell killing, either alone or in combination with anticancer drugs such as cisplatin and docetaxel.

## Conclusions

This study provides the evidence for a role of miR-24-2 in guiding *H2AFX *gene expression in the background of the differential status of gene copy number. Furthermore, the study identifies the antiapoptotic gene *BCL-2 *as a novel cellular target of miR-24-2 and thereby provides a mechanistic insight into the apoptotic induction caused by miR-24-2 overexpression in mammalian cells. We propose that miR-24-2 alone or in combination with anticancer drugs holds strong potential for therapeutic killing of cancer cells.

## Abbreviations

CNA: copy number alteration; CNVs: copy number variations; DDR: DNA damage response; FACS: fluorescence-activated cell sorting; miR: microRNA.

## Authors' contributions

NS participated in the study design, performed laboratory work and statistical analyses and wrote the manuscript. SM performed laboratory work and provided critical comments on the manuscript. AS performed cell culture, its maintenance and fluorescence-activated cell sorting analysis for the study. RP collected laboratory data on the patients and performed the laboratory work and statistical analyses. KP performed the bioinformatics analysis for the study. SC performed the laboratory work for the study. SG provided critical revision of the manuscript and participated in the study design. RD performed the confocal image capturing for the study. RNKB participated in the study design, facilitated the execution of the study and provided critical input in revising the manuscript. All authors read and approved the final manuscript.

## Supplementary Material

Additional file 1 **Supplementary tables**. **Table S1. **Bioinformatics prediction of microRNA targeting *H2AFX *transcript. **Table S2**. Normalization factor for each tissue sample calculated using geNorm software and the normalized expression values for H2AX. **Table S3**. Primer sequence for cloning the predicted miR-24-2 binding site in pGL3 vector. Click here for file

Additional file 2 **Supplementary figures**. **Figure S1**. Expression analysis of microRNA **(a) **hsa-miR-145 and **(b) **hsa-miR-24-2 in sporadic breast cancer samples. **Figure S2**. TaqMan real-time confirmation of overexpression of miR-24-2 in MCF-7 cells after transfection with different concentration of precursor miR-24-2 oligonucleotides (10, 25 and 50 nmol/l). RNU 44 as an endogenous control shows amplification at same cycle threshold value. **Figure S3**. Bioinformatics analysis of miR-24-2 binding sites in transcripts of *H2AFX, BCL-2 *and *MDM2 *genes. **Figure S4**. Fluorescence-activated cell sorting (FACS) analysis of annexin V-stained MCF-7 cells treated with cisplatin (200 mmol/l). **Figure S5**. FACS analysis of annexin V-stained MCF-7 cells treated with H_2_O_2 _(25 mmol/l). **Figure S6. **Luciferase expression in HepG2 cells overexpressing miR-24-2 and transfected with pGL3 control vector or vector harboring the predicted miR-24-2 binding site present in 3'UTR of *H2AFX/BCL-2 *genes. **Figure S7. **Downregulation of miR-24-2 in MCF-7 cells increases the expression of **(a) ***H2AFX *and **(b) ***BCL2 *genes. **Figure S8. **Comparison of miR-24-2 overexpression at 48 hours and 72 hours posttransfection with pre-miR-24-2 (50 nmol/l). Click here for file
