# Supplementary material for: miR-24-2 controls H2AFX expression regardless of gene copy number alteration and induces apoptosis by targeting antiapoptotic gene BCL-2: a potential for therapeutic intervention
Source: Breast Cancer Res. 2011 Apr 4;13(2):R39. doi: 10.1186/bcr2861 (PMC3219202; doi:10.1186/bcr2861)
Supplement: Additional file 1 — Supplementary tables. Table S1. Bioinformatics prediction of microRNA targeting H2AFX transcript. Table S2. Normalization factor for each tissue sample calculated using geNorm software and the normalized expression values for H2AX. Table S3. Primer sequence for cloning the predicted miR-24-2 binding site in pGL3 vector. [file bcr2861-S1.DOC]

**Table S1. Bioinformatics prediction of micro-RNA targeting H2AFX trasncript**

| **Prediction tools** | **No. of microRNA targets in H2AFX transcript** | **Name of the micro RNAs** | **microRNA with a top Score** |
| --- | --- | --- | --- |
| miRanda | 11 | miR-24, miR-938, miR-198, miR-1324, miR-455-3p, miR-1182, miR-484, miR-663b, miR-193a-5p, miR-328, miR-623 | hsa-miR-24 |
| microCosm Targets | 29 | miR-24, miR-650, miR-29c*, miR-520a-3p, miR-373, miR-760, miR-520d-3p, miR-302c, miR-520b,miR-520e, miR-520f, miR-520c-3p, miR-198, miR-938, miR-29a*, miR-302a, miR-219-1-3p, miR-615-5p, miR-302d, miR­182*, miR-18b*, miR-622, miR-675,miR-604,miR-455­5p, miR-937, miR-668,miR-139-3p, miR-339-3p, | hsa-miR-24 |
| PicTar | 2 | miR-24, miR-328 | hsa-miR-24 |
| TargetScan | 2 | miR-24, miR-145 | hsa-miR-24 |

| **TABLE S2** | | | | | |
| --- | --- | --- | --- | --- | --- |
| **PATIET'S AME** | **STAGE** | **ORMALIZATIO FACTOR** | **H2AX (Ct value)** | **H2AX RAW** | **H2AX NORMALISED** |
| PRAMILA GUPTA | 2B | **0.6781** | 25.859 | 0.004083438 | 0.006021692 |
| RITA KUMARI | 2B | **1.5048** | 23.966 | 0.015166164 | 0.01007822 |
| NAJMA AFREEN | 2A | **12.6812** | 19.74 | 0.283810526 | 0.022380477 |
| MRS PRABHA | 2A | **6.5415** | 17.923 | 1 | 0.152870782 |
| PRABHA SUKHLA | 2A | **6.1246** | 19.829 | 0.266831335 | 0.043567152 |
| NEELAM |  |  |  |  |  |
| NIJHWAN | 2B | **8.3375** | 20.264 | 0.197373472 | 0.023672971 |
| MADHU MANGLA | 3A | **1.4237** | 20.531 | 0.164026407 | 0.115213731 |
| SHAHEEDA | 3A | **3.0729** | 20.162 | 0.211833109 | 0.068935197 |
| SUNERI DEVI | 2A | **0.0265** | 26.729 | 0.00223424 | 0.084458344 |
| USHA DEVI | 3B | **7.2331** | 22.492 | 0.042130242 | 0.00582464 |
| NANDINI |  |  |  |  |  |
| MATHUR | 3A | **4.1463** | 27.404 | 0.001399377 | 0.000337502 |
| PRITAM KAUR | 2A | **11.1148** | 24.067 | 0.014140726 | 0.001272244 |
| SACHI SHARMA | 2A | **0.1395** | 29.28 | 0.000381243 | 0.002733789 |
| LAXMI SINGHAL | 2B | **3.5343** | 25.752 | 0.004397807 | 0.001244306 |
| KRISHNA JAIN | 2A | **1.1834** | 28.393 | 0.000705044 | 0.000595795 |
| BHAGWANTI | 3B | **0.1256** | 30.818 | 0.000131286 | 0.001045394 |
| MAYA DEVI JAIN | 2A | **0.4232** | 28.617 | 0.00060365 | 0.001426512 |
| SARLA | 3A | **0.0898** | 31.751 | 6.87634E­05 | 0.000765387 |
| MUMTAZ BEGUM | 2B | **0.1293** | 29.525 | 0.000321699 | 0.0024885 |
| HEMLATA |  |  |  |  |  |
| SHARMA | 2B | **0.1206** | 30.69 | 0.000143466 | 0.001189382 |
| ANITA JAIN | 2B | **0.2492** | 29.65 | 0.000294999 | 0.001183629 |
| MONI BAJPAI | 2B | **1.2146** | 29.699 | 0.000285148 | 0.00023477 |
| PUSHPA |  |  |  |  |  |
| AGARWAL | 2B | **7.4302** | 25.506 | 0.005215423 | 0.000701925 |
| DAYAL KUMARI | 3A | **5.5655** | 25.391 | 0.005648173 | 0.001014857 |
| REKHA GHOSH | 1.000 | **1.9335** | 26.595 | 0.002451703 | 0.001268026 |
| SUKHJEET KAUR | 3B | **1.0717** | 29.218 | 0.000397985 | 0.000371345 |
| PUSHPA DHOOT | 3A | **1.6304** | 26.916 | 0.001961856 | 0.001203277 |
| MONA GUPTA | 1.000 | **3.3314** | 25.628 | 0.004791689 | 0.001438323 |
| RADHA MEHTA | 2A | **5.9304** | 24.573 | 0.009953282 | 0.001678363 |
| ANURADHA |  |  |  |  |  |
| GUPTA | 2B | **2.2154** | 27.275 | 0.001530187 | 0.000690713 |
| PRIYA AGARWAL | 1.000 | **0.2764** | 30.743 | 0.000138201 | 0.00050007 |
| MEENA JAIN | 2B | **0.1822** | 31.2199 | 9.93595E­05 | 0.000545268 |
| SUSHEELA SINGH | 3A | **0.0223** | 31.06 | 0.000110984 | 0.004975334 |
| DALJIT WALIA | 2A | **2.0586** | 26.85 | 0.002054155 | 0.000997849 |
| CANCHAL SINGH | 3B | **4.1508** | 26.026 | 0.003637014 | 0.00087622 |
| GYANWATI | 3A | **0.0154** | 33.721 | 1.75521E­05 | 0.001140266 |

**Table S3** Primer sequence for cloning the predicted miR­24­2 binding site into the pGL3 vector

| Gene Name | Forward Primer | Reverse Primer |
| --- | --- | --- |
| H2AX | 5'­AATCTAGATCCCTTCCAGCAAACTCAAC­3' | 5'­AATCTAGAAACTCCCCAATGCCTAAGGT­3' |
| BCL2 | 5'­AATCTAGACGCATCAGGAAGGCTAGAGT­3' | 5'­AATCTAGAAGCTTCCAGACATTCGGAGA­3' |

*underlined nucleotide sequence are Xba­I restriction digestion site.
