# Supplementary figures and images for: miR-24-2 controls H2AFX expression regardless of gene copy number alteration and induces apoptosis by targeting antiapoptotic gene BCL-2: a potential for therapeutic intervention
Source: Breast Cancer Res. 2011 Apr 4;13(2):R39. doi: 10.1186/bcr2861 (PMC3219202; doi:10.1186/bcr2861)

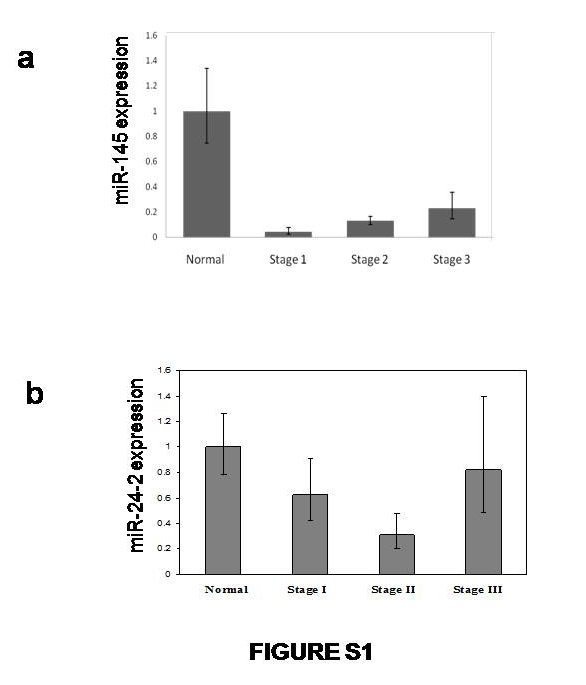


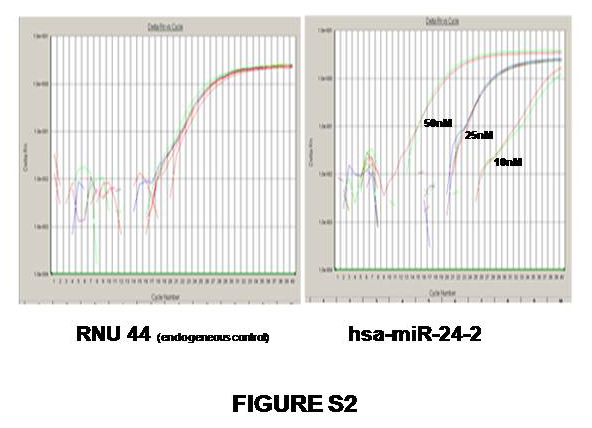


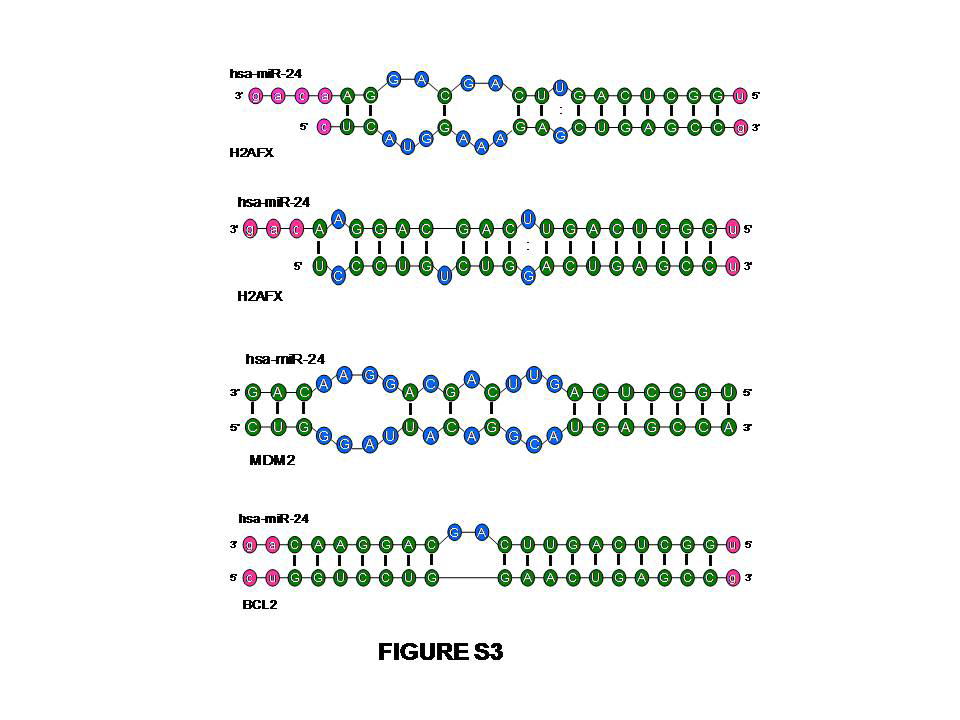


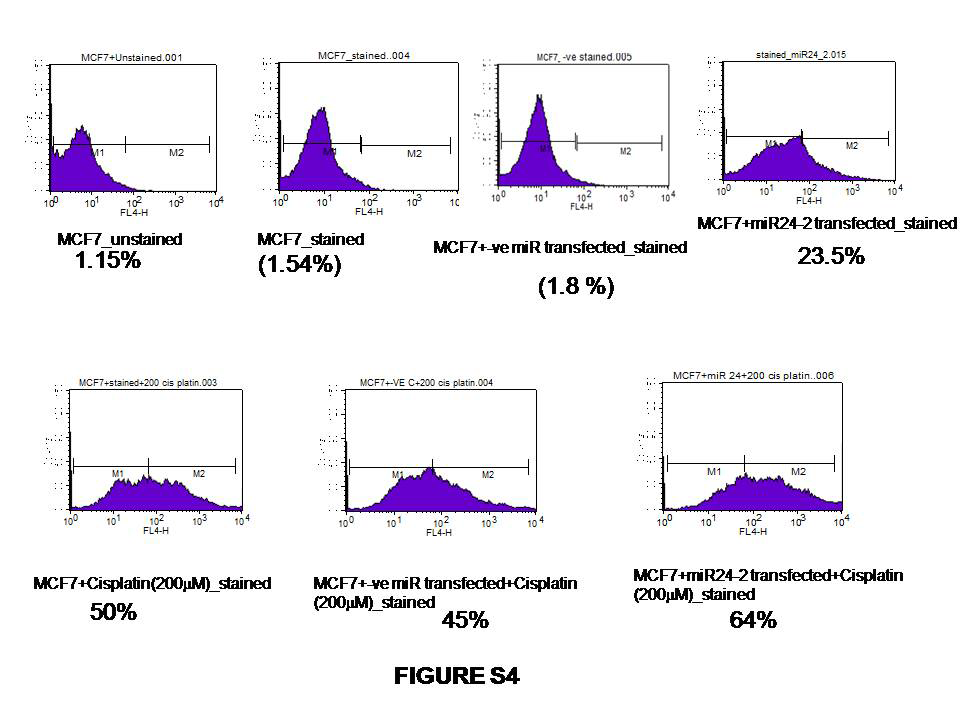


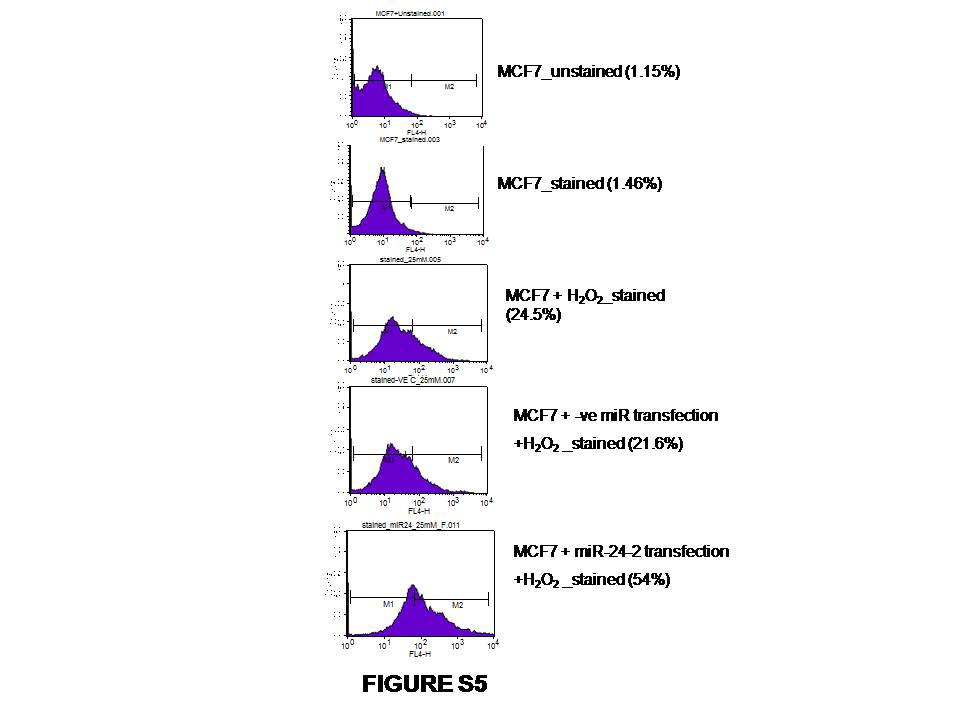


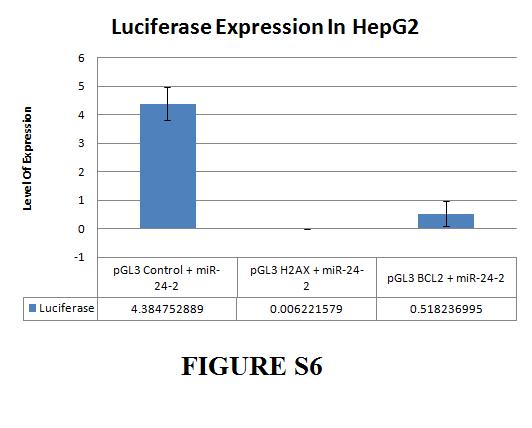


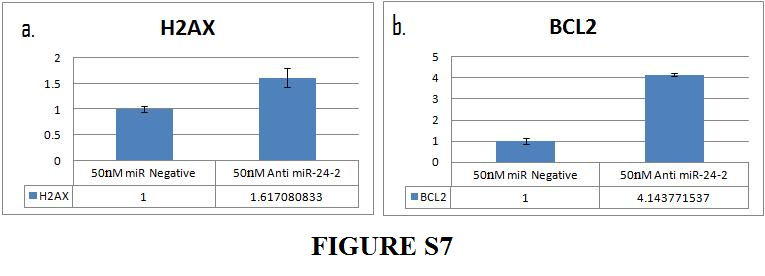


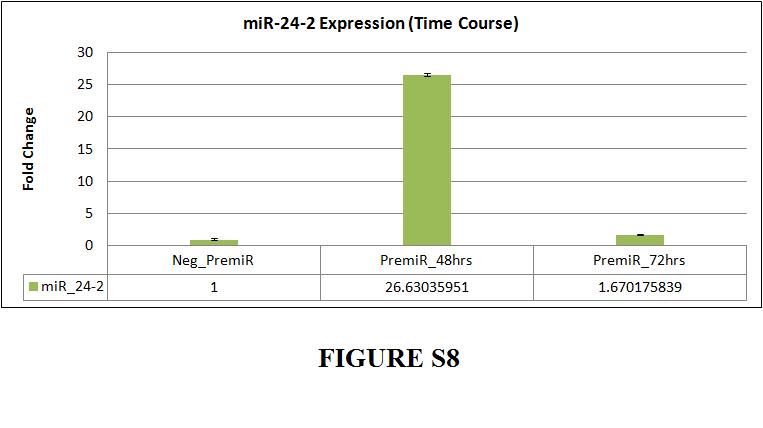

Supplement: Additional file 2 — Supplementary figures. Figure S1. Expression analysis of microRNA (a) hsa-miR-145 and (b) hsa-miR-24-2 in sporadic breast cancer samples. Figure S2. TaqMan real-time confirmation of overexpression of miR-24-2 in MCF-7 cells after transfection with different concentration of precursor miR-24-2 oligonucleotides (10, 25 and 50 nmol/l). RNU 44 as an endogenous control shows amplification at same cycle threshold value. Figure S3. Bioinformatics analysis of miR-24-2 binding sites in transcripts of H2AFX, BCL-2 and MDM2 genes. Figure S4. Fluorescence-activated cell sorting (FACS) analysis of annexin V-stained MCF-7 cells treated with cisplatin (200 mmol/l). Figure S5. FACS analysis of annexin V-stained MCF-7 cells treated with H2O2 (25 mmol/l). Figure S6. Luciferase expression in HepG2 cells overexpressing miR-24-2 and transfected with pGL3 control vector or vector harboring the predicted miR-24-2 binding site present in 3'UTR of H2AFX/BCL-2 genes. Figure S7. Downregulation of miR-24-2 in MCF-7 cells increases the expression of (a) H2AFX and (b) BCL2 genes. Figure S8. Comparison of miR-24-2 overexpression at 48 hours and 72 hours posttransfection with pre-miR-24-2 (50 nmol/l). [file bcr2861-S2.DOC]
